# A Combined Set of Four Serum Inflammatory Biomarkers Reliably Predicts Endoscopic Disease Activity in Inflammatory Bowel Disease

**DOI:** 10.3389/fmed.2019.00251

**Published:** 2019-11-05

**Authors:** Arno R. Bourgonje, Julius Z. H. von Martels, Ruben Y. Gabriëls, Tjasso Blokzijl, Manon Buist-Homan, Janette Heegsma, Bernadien H. Jansen, Hendrik M. van Dullemen, Eleonora A. M. Festen, Rinze W. F. ter Steege, Marijn C. Visschedijk, Rinse K. Weersma, Paul de Vos, Klaas Nico Faber, Gerard Dijkstra

**Affiliations:** ^1^Department of Gastroenterology and Hepatology, University Medical Center Groningen, University of Groningen, Groningen, Netherlands; ^2^Department of Laboratory Medicine, University Medical Center Groningen, University of Groningen, Groningen, Netherlands; ^3^Department of Gastroenterology and Hepatology, Martini Hospital, Groningen, Netherlands; ^4^Department of Pathology and Medical Biology, University Medical Center Groningen, University of Groningen, Groningen, Netherlands

**Keywords:** Inflammatory Bowel Disease (IBD), inflammatory biomarkers, endoscopy, inflammation, disease activity

## Abstract

**Introduction:** Blood C-reactive protein (CRP) and fecal calprotectin levels are routinely measured as surrogate markers of disease activity in Inflammatory Bowel Disease (IBD), but often do not correlate well with the degree of mucosal inflammation in the intestine as established by endoscopy. Therefore, novel predictive biomarkers are urgently needed that better reflect mucosal disease activity in IBD. The aim of this study was to identify a combination of serum inflammatory biomarkers predictive for endoscopic disease activity.

**Methods:** Serum concentrations of 10 inflammatory biomarkers were analyzed in 118 IBD patients [64 Crohn's disease (CD), 54 ulcerative colitis (UC)] and 20 healthy controls. In a subset of 71 IBD patients, endoscopic disease activity was established. Non-parametric ROC estimation with bootstrap inference was used to establish the best combination of inflammatory biomarkers predicting endoscopic disease activity.

**Results:** Six (6) inflammatory biomarkers (serum amyloid A (SAA), Eotaxin-1, IL-6, IL-8, IL-17A, and TNF-α) showed better prediction of IBD disease activity than routine measures (CRP, fecal calprotectin and HBI/SCCAI scores). The best combination of predictive inflammatory biomarkers consisted of serum SAA, IL-6, IL-8, and Eotaxin-1, showing an optimism-adjusted area under the ROC (AuROC) curve of 0.84 (95% CI: 0.73–0.94, *P* < 0.0001), which predicted significantly better (*P* = 0.002) than serum CRP levels with an AuROC of 0.57 (95% CI: 0.43–0.72, *P* = 0.32).

**Conclusion:** The combination of SAA, IL-6, IL-8, and Eotaxin-1 reliably predicts endoscopic disease activity in IBD and might be valuable for monitoring disease activity and management of the disease.

## Introduction

Crohn's disease (CD) and ulcerative colitis (UC) are chronic idiopathic inflammatory bowel diseases (IBD), characterized by an inappropriate and uncontrolled immune response, stimulated by the gut microbiome in a genetically susceptible host (1). Typically, patients with IBD follow a disease course consisting of alternating exacerbations and periods of remission (2). In IBD, the extent of inflammatory disease activity is preferably established by endoscopy, that is translated to validated scoring systems, such as the Mayo endoscopic subscore for UC and the Simple Endoscopic Score for CD (SES-CD) (3, 4). Frequent monitoring of IBD disease activity is crucially important, since long-lasting subclinical disease activity is known to increase the risk of future surgical interventions and hospitalization and to decrease patients' quality of life and economic participation (5–8). Endoscopic examination is still the most reliable approach for diagnosing the presence and extent of IBD disease activity (9). This procedure, however, has several disadvantages, such as a high patient burden, but also risks of serious complications, like bowel perforation or bleeding. In addition, it is costly and time-consuming. Alternatives for endoscopy are therefore urgently needed.

Non-endoscopic disease indices, such as the Crohn's Disease Activity Index (CDAI) or Harvey Bradshaw Index (HBI) for CD and the Simple Clinical Colitis Activity Index (SCCAI) for UC, fail to correlate well with endoscopically-proven intestinal inflammation (10–13). Biomarkers for endoscopic disease activity have also been explored and are becoming increasingly important to predict the level of mucosal inflammation in IBD. Fecal calprotectin (FC) and serum C-reactive protein (CRP) levels are now widely used and considered predictive markers for the degree of inflammation, but also show inconsistent correlation with mucosal inflammation when compared to endoscopy (14–16). This illustrates the need for better diagnostic measures for IBD exacerbations that preferably can also be applied to patients with subclinical disease activity (17).

Cytokines play a pivotal role in the pathogenesis of IBD, controlling intestinal inflammation and disease activity, and might be better predictive markers for disease activity than FC and CRP (18–20). In many diseases, combinations of inflammatory cytokines have been shown to be predictive for inflammatory state and are therewith adequate biomarkers for non-invasive disease activity monitoring (21). Recently, we showed that for CD a positive correlation exists between multiple Th1- and Th17-associated serum cytokines and fecal calprotectin levels (22). Although no endoscopic results were available for that patient cohort, it demonstrated the proof of principle and value of selected Th1- and Th17-associated serum cytokines for measuring inflammation in IBD. As a next step, we aimed to evaluate the potential of a combined set of such cytokines to predict endoscopic disease activity, as one single biomarker will be unlikely to accurately predict the mucosal status in IBD. Therefore, a limited set of 10 candidate inflammatory biomarkers (C-reactive protein (CRP), serum amyloid A (SAA), IFN-γ, TNF-α, IL-6, IL-8, IL-10, IL-17A, Eotaxin-1, and Eotaxin-3) was selected based on results from the aforementioned pilot-study and available literature (18, 20–22).

In the present study, we investigated a selection of 10 inflammatory biomarkers involved in IBD and their association to inflammatory disease activity as evaluated by endoscopic examination. Correlations between individual biomarkers and endoscopic disease activity were analyzed and used to compose an accurate prediction tool for the level of endoscopic disease activity, based on a subset of these biomarkers. Finally, we compared the predictive accuracy of this panel of biomarkers with commonly applied measures of disease activity, such as clinical indices (HBI/SCCAI), serum CRP, and FC levels.

## Materials and Methods

### Study Population

This cohort study included patients from the database of the IBD center of the University Medical Center Groningen (UMCG). Serum samples from 118 IBD patients, either CD (*n* = 64) or UC (*n* = 54) were collected. At the moment serum samples were obtained, all patients had an indication for starting new biological therapy. Indications to initiate biological therapy were primarily based on increased endoscopic disease activity, though few (CD) patients had fistulizing disease as therapy indication. Inclusion criteria for this study were: age ≥ 18 years and an established diagnosis of IBD existing for at least 1 year. Diagnosis was based on clinical, endoscopic and histological criteria (23). Clinically relevant data were retrieved from the patients' medical records: age, gender, body-mass index (BMI), smoking status, Montreal classification, maintenance medication (mesalamine, thiopurines, methotrexate, TNF-antagonists), disease duration, previous anti-TNF therapy and surgical history. Clinical disease activity was recorded by scoring the Harvey Bradshaw Index (HBI) for CD and the Simple Clinical Colitis Activity Index (SCCAI) for UC (24, 25).

### Ethical Considerations

Serum samples were obtained after patients gave written informed consent (study approved by the Institutional Review Board [IRB] of the UMCG registered as no. 08/338). In addition, serum samples of 20 healthy controls were included for comparison. These serum samples were retrieved from an UMCG biobank containing pre-donation samples of living kidney donors (PSI-UMCG [IRB no. 08/279]). This study has been performed in accordance with the principles of the Declaration of Helsinki (2013).

### Laboratory Measurements

Serum samples for measurements of routine diagnostic laboratory parameters, including hemoglobin levels, C-reactive protein (CRP) levels, erythrocyte sedimentation rate (ESR), white blood cell count (WBC) and thrombocyte counts, were obtained simultaneously with the serum samples collected for measurements of detected inflammatory biomarkers. At the same time-point, fecal calprotectin levels were quantified by enzyme-linked immunosorbent assays (ELISA) (Bühlmann Laboratories AG, Switzerland) in a subgroup of patients (*n* = 25).

### Endoscopic Disease Activity

Baseline endoscopy investigation was performed in a subset of 71 IBD patients (CD, *n* = 36; UC, *n* = 35) within 3 months prior to serum sample collection (median (IQR) interval: 31 [19–60] days). Endoscopic disease activity was graded based on available endoscopic images and endoscopy reports written by certified gastroenterologists from our university hospital who were not involved in the study. Disease activity was scored according to the validated Simplified Endoscopic Score for CD (SES-CD) and Mayo endoscopic subscore for UC (3, 4). To calculate the SES-CD, 5 different bowel segments were scored and defined as follows: ileum (excluding the ileocecal valve or ileocolonic anastomosis), ascending colon (including ileocecal valve, cecum, and ascending colon until the hepatic flexure), transverse colon (between hepatic and splenic flexures), descending colon (from splenic flexure to rectosigmoid junction) and rectum. All 5 segments were evaluated for 4 different endoscopic variables scored from 0 to 3: size of ulcers, ulcerated surface, affected surface, and the presence of narrowings. Ultimately, SES-CD scores were defined as previously described: endoscopic remission 0–3 points (category 0), mild disease activity 4–10 points (category 1), moderate disease activity 11–19 points (category 2) and severe disease activity ≥20 points (category 3) (26). For UC, the Mayo endoscopic subscore for endoscopic disease activity was obtained from endoscopy reports written by certified gastroenterologists. Here, Mayo 0 was defined as endoscopic remission (normal mucosa), Mayo 1 as mild disease activity (erythema, decreased vascular pattern, mild friability), Mayo 2 as moderate disease activity (marked erythema, lack of vascular pattern, friability, erosions) and Mayo 3 as severe disease activity (spontaneous bleeding and ulceration). For the purpose of analysis, categories from both endoscopy scores of CD (SES-CD) and UC (Mayo endoscopic subscore) were merged on categorical level of mucosal damage (0–3) to finally create an IBD composite endoscopy score (27).

### Measurement of Inflammatory Biomarkers

A selection of 10 inflammatory biomarkers were measured based on a previously performed study and available literature (22). In short, serum samples from all subjects were collected and stored in 1 mL aliquots at −80°C. After thawing and prior to analysis, samples were centrifuged for 3 min at 2,000 g to remove remaining debris. Measurement of serum levels of C-reactive protein (CRP), serum amyloid A (SAA), IFN-γ, TNF-α, IL-6, IL-8, IL-10, IL-17A, Eotaxin-1, and Eotaxin-3 was implemented using a customized electrochemiluminescence (ECL) multiplex assay (Meso Scale Discovery (MSD®), Meso Scale Diagnostics, Rockville, MD). ECL signals were fitted to a 4-parameter logistic model with 1/y^2^ weighting, ensuring a broad and dynamic range of molecule detection. Serum concentrations of all detected molecules were determined by using calibration curves to which the ECL signals were back-fitted. Final concentrations were calculated using the MSD Discovery Workbench analysis software®. Of all detected biomarker concentrations, 94.0% of values were within the detection range and remaining values (6.0%) were excluded from further analysis.

### Statistical Analysis

Baseline demographic and clinical characteristics were presented as means ± standards errors (SEM) or proportions with corresponding percentages (*n*, %). Serum concentrations of inflammatory biomarkers were presented as median ± interquartile ranges (IQR). Assessment of normality of continuous variables was performed using normal Q-Q plots. Continuous variables were compared using Student's *t*-tests or Mann-Whitney *U*-tests according to normality. Categorical variables were compared using chi-square tests or Fisher's exact test, as appropriate. All consecutive analyses were performed in the subset of 71 IBD patients with available endoscopic results within 3 months prior to serum analysis. Simple correlations between inflammatory biomarkers and measures of disease activity were established using the non-parametric Spearman's correlation coefficient (ρ). To evaluate predictive performance of all detected inflammatory biomarkers regarding composite IBD endoscopic disease activity, receiver operating characteristics (ROC) curves were established with associated areas under the ROC curve (AuROCs) as overall measure of fit. ROC curves and associated AuROCs were established using the non-parametric, tie-corrected trapezoidal approximation method. Two correlated areas under the ROC curve were compared with each other using a non-parametric approach based on properties from generalized *U*-statistics to estimate a covariance matrix (28). Optimal thresholds for the most promising serum inflammatory biomarkers (serum amyloid A (SAA), Eotaxin-1, IL-6, IL-8, IL-17A, and TNF-α) were determined by equally maximizing sensitivity and specificity to compute the Youden's index (*J* statistic). Optimal thresholds or cut-off points (*c*) were established by selecting the highest Youden's index, defined as:

$$\begin{array}{l} J={\text{max}}_{c}\;\left\{sensitivity\left(c\right)+specificity\left(c\right)-1\right\} \end{array}$$

Combinations of classifiers were empirically tested for their predictive performance using a non-parametric ROC estimation of combined predicted probabilities (derived from multivariable logistic regression) with bootstrap inference. Data were analyzed using SPSS Statistics 23.0 software package (SPSS Inc., Chicago, ILL, USA) and STATA software (version 15.0, Stata Corp, College Station, Texas, USA; commands used: “roctab,” “roccomp,” and “rocreg”) and visualized using GraphPad Prism version 6.0 (La Jolla, CA, USA). In case of multiple testing, Bonferroni corrections were applied. Two-tailed *P* ≤ 0.05 were considered as statistically significant.

### Internal Validation

Because all biomarker performances were tested on the same dataset, AuROCs and Youden's indices as overall measures of predictive performance could potentially be overestimated due to the correlated nature of the data. To adjust for this potential bias, a bootstrap resampling procedure using 20,000 replicates was performed as internal validation and to obtain standard errors (SE) and confidence intervals (CI) for the AuROCs of best biomarker combinations.

## Results

### Study Cohort Characteristics

Baseline demographic and clinical characteristics of the total study population (*n* = 138) are presented in Supplementary Table 1. The IBD study cohort consisted of 118 patients, of which 64 patients with CD and 54 patients with UC. For comparison, 20 healthy individuals (healthy controls, HC) were included in the study. IBD patients had a significantly lower mean age (CD: 43.8 ± 1.8 years; UC: 47.0 ± 2.0 years) as compared to healthy controls (56.1 ± 2.2 years), while no significant gender differences were observed [CD: 39 females (60.9%); UC: 26 females (48.1%); HC: 12 females (60.0%)]. Further differences between CD and UC patients were largely related to disease-specific characteristics (Supplementary Table 1).

For all IBD patients, different measures of disease activity were recorded and compared between CD and UC patients (Supplementary Table 2). As clinical disease activity index, the Harvey Bradshaw Index (HBI) was calculated for CD patients, whereas the Simple Clinical Colitis Activity Index (SCCAI) was recorded for UC patients. Median HBI score was 8 points (IQR: 6–11) and median SCCAI score was 6 points (IQR: 4–8). Serum CRP levels and ESR (mm/h) were significantly higher in CD patients as compared to UC patients, whereas the latter group showed significantly higher levels of FC. Considering endoscopic disease activity, more CD patients fell into either remission (0–3 points) or mild (4–10 points) disease categories (CD: 47.2%; UC: 20.0%), whereas the majority of UC patients belonged to moderate (11–19 points) and severe (≥20 points) disease categories (UC: 80.0%; CD: 52.8%).

### Analysis of 10 Inflammatory Biomarkers in 118 IBD Patients and 20 Healthy Controls

**Figure 1:**
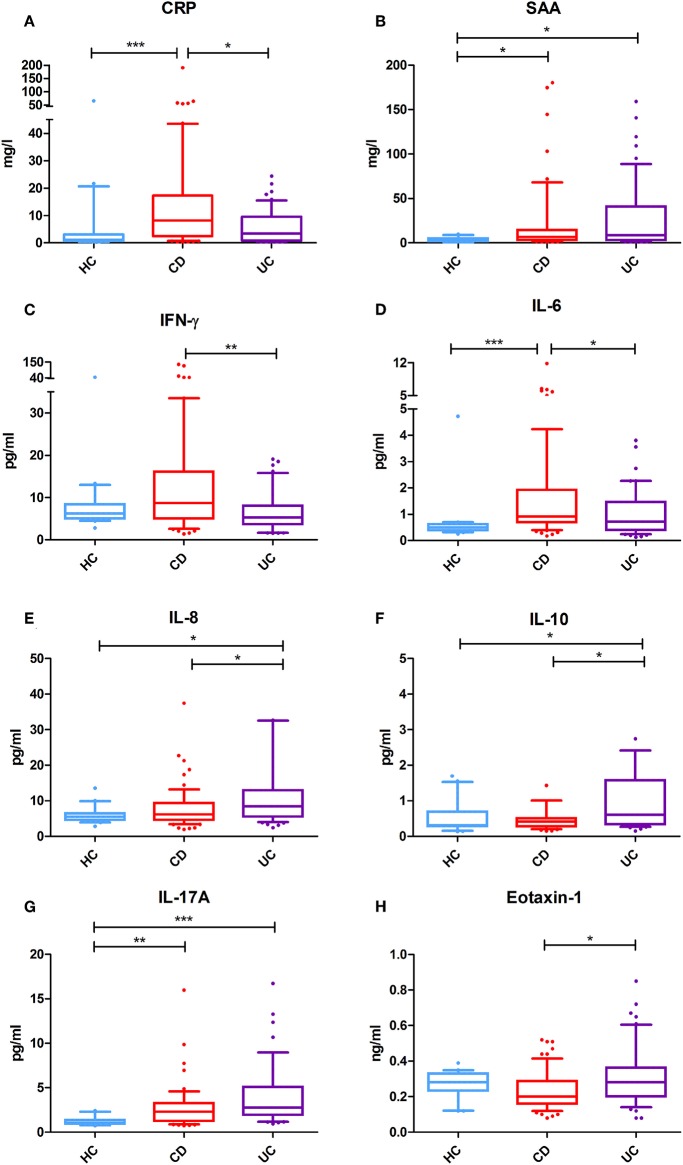
Serum levels of selected inflammatory biomarkers in Crohn's disease (CD) (*n* = 64) and ulcerative colitis (UC) (*n* = 54) patients and healthy controls (HC) (*n* = 20). **(A)** Serum CRP levels (mg/l) are significantly increased in CD as compared to UC and healthy controls. **(B)** Serum SAA levels (mg/l) are significantly increased in IBD as compared to healthy controls. **(C)** Serum IFN-γ levels (pg/ml) are significantly more elevated in CD as in UC. **(D)** Serum IL-6 levels (pg/ml) are significantly increased in CD as compared to UC and healthy controls. **(E)** Serum IL-8 levels (pg/ml) are specifically more elevated in UC as compared to CD and HC. **(F)** Serum IL-10 levels (pg/ml) are also significantly increased in UC as compared to CD or HC. **(G)** Serum IL-17A levels (pg/ml) are strongly significantly increased in both CD and UC as compared to HC. **(H)** Serum Eotaxin-1 levels (ng/ml) are significantly elevated in UC as compared to CD, but comparable with that of HC. *P < 0.05 were considered statistically significant. **P < 0.01. ***P < 0.001.

Serum concentrations of 10 selected serum inflammatory biomarkers in IBD patients and healthy controls are presented in Table 1. In CD patients, four (4) out of 10 inflammatory biomarkers (CRP, SAA, IL-6, and IL-17A) showed significantly increased concentrations as compared to healthy controls (HC). Also, four (4) out of 10 biomarkers were significantly increased in UC compared to HC (SAA, IL-8, IL-10, and IL-17A), where SAA and IL-17A overlapped with CD. In addition, the levels of 6 inflammatory biomarkers were significantly different between CD and UC patients: levels of CRP, IFN-γ, and IL-6 were significantly higher in CD, while IL-8, IL-10, and Eotaxin-1 levels were significantly higher in UC (Figure 1). No significant differences were observed for serum levels of TNF-α and Eotaxin-3 between CD, UC, and HC.

**Table 1:** Median (IQR) of baseline serum concentrations of all detected molecules in CD (*n* = 64) and UC (*n* = 54) patients as compared to healthy controls (HC) (*n* = 20).

**Detected molecules**	**CD**	**UC**	**HC**	***P*-value**
CRP (mg/l)	8.17 (2.42–17.3)	3.37 (0.86–9.48)	1.11 (0.71–3.08)	**<0.001**
SAA (mg/l)	6.53 (3.31–14.5)	8.75 (2.85–40.9)	3.41 (1.67–5.12)	**0.005**
IFN-γ (pg/ml)	8.68 (5.03–16.1)	5.29 (3.67–8.04)	6.23 (5.03–8.40)	**0.007**
TNF-α (pg/ml)	2.15 (1.71–2.84)	2.29 (1.42–3.39)	2.12 (1.81–2.47)	0.578
IL-6 (pg/ml)	0.91 (0.69–1.92)	0.72 (0.40–1.46)	0.49 (0.38–0.62)	**<0.001**
IL-8 (pg/ml)	6.16 (4.62–9.36)	8.42 (5.51–13.0)	5.47 (4.61–6.50)	**0.005**
IL-10 (pg/ml)	0.41 (0.28–0.51)	0.61 (0.34–1.58)	0.31 (0.22–0.41)	**0.004**
IL-17A (pg/ml)	2.30 (1.24–3.26)	2.76 (1.94–5.07)	1.04 (0.94–1.36)	**<0.001**
Eotaxin-1 (ng/ml)	0.20 (0.16–0.29)	0.28 (0.20–0.36)	0.28 (0.23–0.33)	**0.018**
Eotaxin-3 (pg/ml)	17.0 (12.4–23.6)	19.2 (14.5–22.6)	19.6 (13.3–29.6)	0.454

*Data are presented as median (IQR)*.

*Differences between groups were tested using Kruskal-Wallis tests. P < 0.05 were considered statistically significant (Bonferroni-adjusted, indicated in bold)*.

### Correlations of Inflammatory Biomarkers With Endoscopic Disease Activity in IBD

Endoscopic examination of 71 (CD: *n* = 36 and UC: *n* = 35) of the 118 IBD patients was available and this subgroup was used to analyze correlations between the individual serum biomarkers and clinical (HBI/SCCAI), biochemical (CRP, fecal calprotectin) and endoscopic (CD: SES-CD score, UC: Mayo score, IBD: composite endoscopy score) measures of disease activity using Spearman's rank correlation coefficients (ρ). Baseline demographic and clinical characteristics of this subset of patients (*n* = 71) are presented in Supplementary Table 3. Correlations between serum biomarkers and measures of disease activity are presented in a correlation matrix (Table 2). The SES-CD score positively correlated with serum amyloid A (SAA) (ρ = 0.410, *P* < 0.05), closely followed by IFN-γ (ρ = 0.383, *P* < 0.05), IL-8 (ρ = 0.359, *P* < 0.05) and IL-17A (ρ = 0.352, *P* < 0.05), while the Mayo endoscopic subscore (for UC) correlated only significantly with serum levels of IL-6 (ρ = 0.356, *P* < 0.05). An IBD composite endoscopy score was created by merging both endoscopy scores of CD (SES-CD) and UC (Mayo) on categorical level of disease activity (0, 1, 2, or 3). Using this composite IBD endoscopy score (see Supplementary Table 2; *n* = 71), significant correlations were observed for Eotaxin-1 (ρ = 0.316, *P* < 0.01), IL-8 (ρ = 0.295, *P* < 0.05) and SAA (ρ = 0.288, *P* < 0.05) (Supplementary Figure 1). Furthermore, routinely-measured CRP levels (mg/l) correlated significantly with multiple biomarkers analyzed by the ECL multiplex assay (CRP, SAA, IL-6, IFN-γ, and TNF-α). In contrast, fecal calprotectin (FC) levels (*n* = 25) did not show significant correlations with any of the detected inflammatory biomarkers. Similarly, clinical disease indices only showed a significant correlation with serum IL-6 levels (ρ = 0.349, *P* < 0.01), whereas the remaining inflammatory biomarkers did not correlate with either HBI or SCCAI scores.

**Table 2:** Correlations between serum levels of individual biomarkers with endoscopic (SES-CD, Mayo score and composite IBD endoscopy score), biochemical (CRP and fecal calprotectin, FC) and clinical (HBI or SCCAI) measures of disease activity.

	**SES-CD (*n* = 36)**	**Mayo (*n* = 35)**	**Composite (*n* = 71)**	**HBI/SCCAI (*n* = 56)**	**CRP (*n* = 113)**	**FC (*n* = 25)**
CRP (mg/l)	0.155	−0.053	−0.067	0.101	0.871^**^	0.022
SAA (mg/l)	0.410^*^	0.208	0.288^*^	0.006	0.605^**^	0.064
IFN-γ (pg/ml)	0.383^*^	0.119	0.048	0.034	0.325^**^	−0.221
TNF-α (pg/ml)	0.021	0.183	0.175	−0.048	0.298^**^	−0.089
IL-6 (pg/ml)	0.164	0.356^*^	0.129	0.349^**^	0.450^**^	0.079
IL-8 (pg/ml)	0.359^*^	0.118	0.295^*^	−0.076	0.002	0.006
IL-10 (pg/ml)	0.097	−0.023	0.127	0.172	−0.020	0.189
IL-17A (pg/ml)	0.352^*^	−0.073	0.202	−0.125	0.185	0.113
Eotaxin-1 (ng/ml)	0.212	0.144	0.316^**^	0.060	−0.121	0.053
Eotaxin-3 (pg/ml)	−0.205	−0.217	−0.110	−0.059	−0.098	0.028

*Correlation matrix showing Spearman's rank correlation coefficients (ρ) for associations between all detected molecules and clinical, biochemical and endoscopic measures of disease activity*.

* *P < 0.05 were considered statistically significant*.

** *P < 0.01. Significances are indicated in bold*.

### Predicting Endoscopic Disease Activity Using Inflammatory Biomarkers

To test the predictive performances of selected inflammatory biomarkers, distributions of serum concentrations of all biomarkers were compared between IBD patients with binary categorized, composite IBD endoscopic disease activity: remission (0) or mild (1) endoscopic disease activity vs. moderate (2) or severe (3) endoscopic disease activity (Supplementary Table 4). Subsequently, subgroup analyses were performed for CD and UC patients separately, which can be found in Supplementary Table 5 and are visualized in Supplementary Figures 2–5.

**Figure 2:**
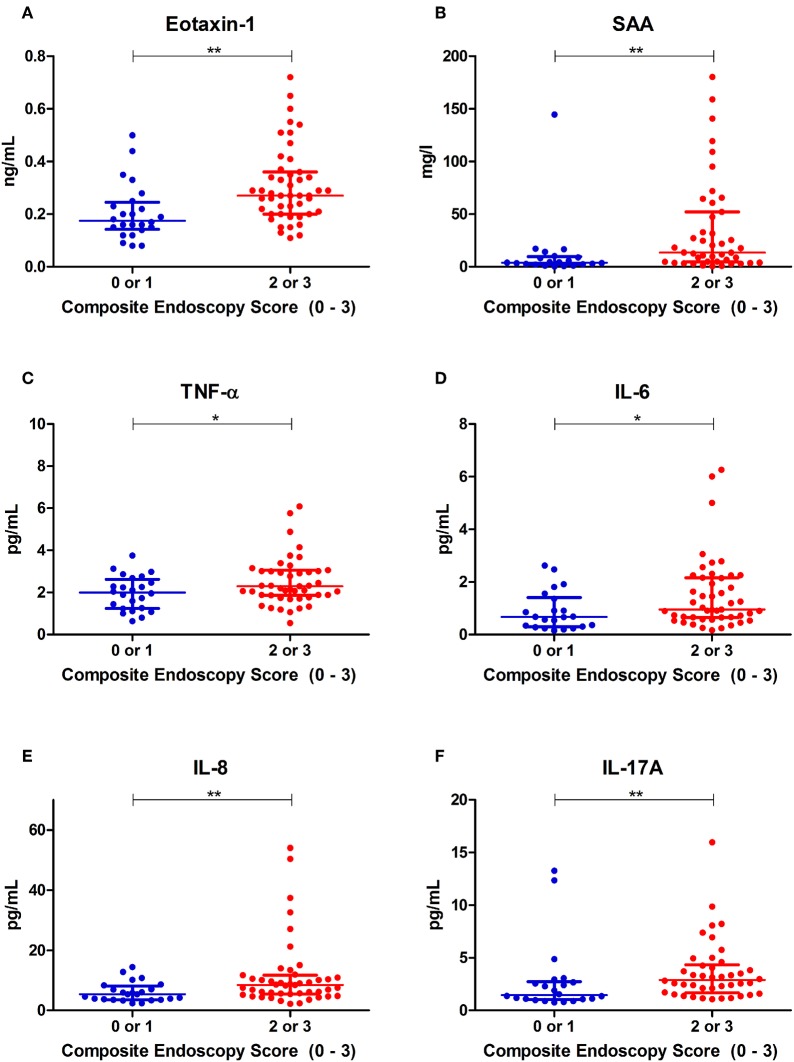
Distributions of serum concentrations of **(A)** Eotaxin-1, **(B)** serum amyloid A (SAA), **(C)** tumor necrosis factor alpha (TNF-α), **(D)** interleukin-6 (IL-6), **(E)** interleukin-8 (IL-8) and **(F)** interleukin-17A (IL-17A), that were significantly different among binary ordered endoscopic disease activity, using a composite IBD endoscopy score (0 or 1 indicating remission or mild disease and 2 or 3 indicating moderate or severe disease, respectively). *P < 0.05. **P < 0.01.

Using the composite IBD endoscopy score, patients with high endoscopic disease activity [either moderate (2) or severe (3)] demonstrated significantly elevated serum concentrations of Eotaxin-1, SAA, TNF-α, IL-6, IL-8, and IL-17A as compared to patients with low endoscopic disease activity [either remission (0) or mild (1)] (Figure 2). In the CD subgroup, using the binary ordered SES-CD, significantly increased concentrations of SAA, IFN-γ, IL-6, and IL-17A were observed in patients with high endoscopic disease activity (Supplementary Table 5; Supplementary Figure 2). In UC, using the binary Mayo endoscopic subscore categories, serum concentrations of IL-6, TNF-α, and Eotaxin-1 were significantly increased in moderate-to-severe disease activity as compared to remission or mild disease activity (Supplementary Table 5; Supplementary Figure 4).

**Figure 3:**
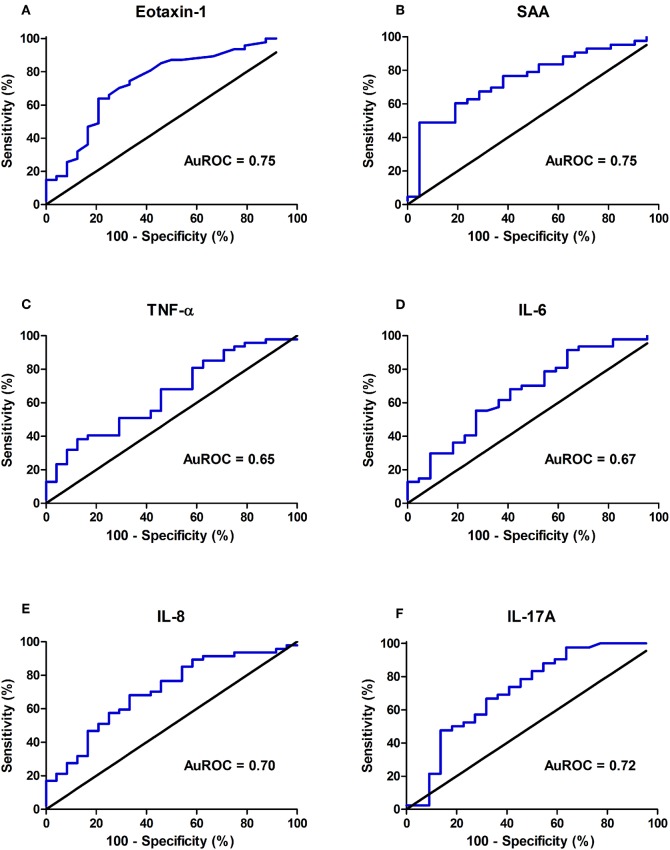
Discriminative capacity of serum concentrations of **(A)** Eotaxin-1, **(B)** serum amyloid A (SAA), **(C)** tumor necrosis factor alpha (TNF-α), **(D)** interleukin-6 (IL-6), **(E)** interleukin-8 (IL-8), **(F)** interleukin-17A (IL-17A) regarding binary ordered endoscopic disease activity (remission (0) or mild (1) disease vs. moderate (2) or severe (3) disease), as represented by the area under the receiver operating characteristics curve (AuROC). Of all individual molecules shown, Eotaxin-1 and SAA display the best discriminative capacity regarding binary ordered endoscopic disease activity.

To evaluate their predictive accuracies with respect to endoscopically active disease, receiver operating characteristics (ROC) curves were established (Figure 3). In the ROC analysis, serum levels of Eotaxin-1 (pg/ml) and SAA (mg/l) presented the best discriminative capacity regarding binary ordered, composite IBD endoscopic disease activity (area under the receiver operating characteristics curve (AuROC) 0.75 (SE: 0.06, 95% CI: 0.62–0.87, *P* < 0.001) for both serum Eotaxin-1 and SAA levels) (Table 3). Serum levels of IL-17A, IL-8, IL-6, and TNF-α were of subordinate, but still reasonable discriminative value.

**Table 3:** ROC analysis showing discriminative power of individual inflammatory biomarkers that are significantly increased in IBD patients with moderate (2) or severe (3) endoscopic disease activity as compared to patients with remission (0) or mild (1) disease activity, as determined by the binary categorized, composite IBD endoscopy score (CD: SES-CD, UC: Mayo endoscopic subscore).

	**AuROC (95% CI)**	**Sensitivity/ Specificity**	**Cut-off value**	**Youden's *J* statistic**
**Inflammatory biomarkers**				
Eotaxin-1 (ng/ml)	0.75 (0.62–0.87)	74.5/66.7%	>0.21 ng/ml	0.41^***^
SAA (mg/l)	0.75 (0.62–0.87)	48.8/95.2%	>17.5 mg/l	0.44^**^
TNF-α (pg/ml)	0.65 (0.52–0.78)	38.3/87.5%	> 2.88 pg/ml	0.26^*^
IL-6 (pg/ml)	0.67 (0.53–0.81)	55.3/72.7%	> 0.91 pg/ml	0.28^*^
IL-8 (pg/ml)	0.70 (0.58–0.83)	68.1/66.7%	> 6.12 pg/ml	0.35^**^
IL-17A (pg/ml)	0.72 (0.57–0.86)	66.7/68.2%	> 2.40 pg/ml	0.35^**^
**Routine measures**				
CRP (mg/l)	0.57 (0.43–0.72)	51.1/66.7%	> 5.73 mg/l	0.18
FC (μg/g)	0.66 (0.44–0.90)	80.1/50.0%	> 735 μg/g	0.32
HBI/SCCAI	0.66 (0.49–0.83)	62.9/64.3%	> 6.5 points	0.27

^*^P-values were calculated for the difference between the area under the ROC curve and the no-discrimination line (AuROC = 0.50).

* *P < 0.05 were considered statistically significant*.

** *P < 0.01*.

*** *P < 0.001*.

### Best Combinations of Inflammatory Biomarkers to Predict Endoscopic Disease Activity

**Figure 4:**
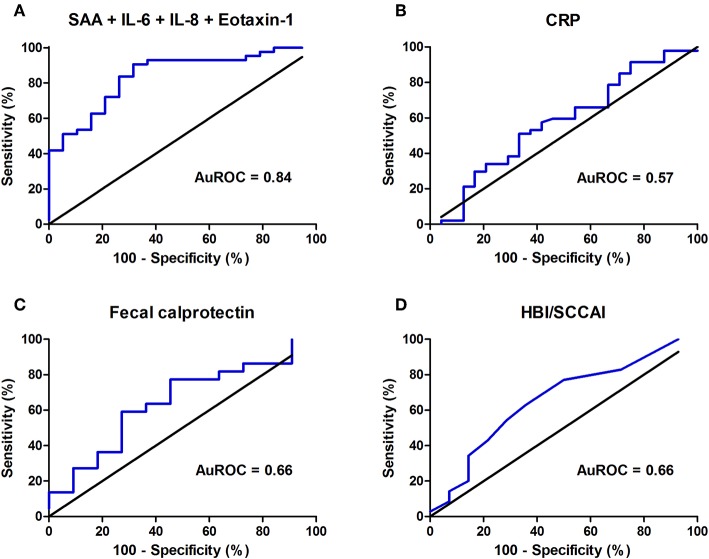
Areas under the receiver operating characteristics curve (AuROC) for **(A)** the best predictive combination of biomarkers (serum amyloid A (SAA), interleukin-6 (IL-6), interleukin-8 (IL-8) and Eotaxin-1) (*n* = 64), **(B)** serum C-reactive protein (CRP) levels, **(C)** fecal calprotectin (FC) levels (μg/g) (*n* = 25) and **(D)** Harvey Bradshaw Index (HBI) or Simple Clinical Colitis Activity Index (SCCAI) (*n* = 56).

To achieve the best discrimination between remission (0) or mild (1) vs. moderate (2) or severe (3) endoscopic disease activity, multiple combinations of detected inflammatory biomarkers were empirically investigated for their predictive accuracy. Ultimately, for the composite IBD endoscopy score, the best predictive combination of inflammatory biomarkers was represented by the assembly of serum levels of SAA, IL-6, IL-8, and Eotaxin-1, showing an AuROC of 0.84 (SE: 0.05, 95% CI: 0.73–0.94, *P* < 0.0001, *n* = 64) (Figure 4A). In this combination, SAA could be replaced by serum CRP levels without losing predictive accuracy (correlation between CRP and SAA: ρ = 0.663, *P* < 0.01) (Supplementary Figure 6). Applying the algorithm for comparison of correlated ROC curves, the AuROC for this combination of biomarkers was significantly better as compared to that of serum CRP levels (*P* = 0.002), whereas no statistical significance emerged when compared to fecal calprotectin levels or the clinical disease scores (HBI/SCCAI) (*P* = 0.313 and *P* = 0.073, respectively). Fecal calprotectin levels closest to the date of endoscopy were incorporated into this analysis (*n* = 25, all within 3 months, median (IQR) time interval 39 [25–55] days). Serum CRP levels had an AuROC of 0.57 (SE: 0.07, 95% CI: 0.43–0.72, *P* = 0.32), fecal calprotectin (FC) levels 0.66 (*n* = 25, SE: 0.12, 95% CI: 0.44–0.90, *P* = 0.17) and HBI or SCCAI scores 0.66 (*n* = 56, SE: 0.09, 95% CI: 0.49–0.83, *P* = 0.08) (Figures 4B–D). The resulting combined calculated probability had a maximum sensitivity of 90.7% and specificity of 68.4% in correctly discriminating IBD patients with low or high endoscopic disease activity (Youden's *J* statistic = 0.58).

In the CD subgroup, regarding the predictive value for SES-CD scored endoscopic disease activity, serum levels of SAA presented the best discriminative capacity as represented by an AuROC of 0.79 (SE: 0.09, 95% CI: 0.61–0.96, *P* < 0.01) (Supplementary Figure 7). In the UC subgroup, the combination of IL-6 and Eotaxin-1 demonstrated the best predictive performance (AuROC 0.97, SE: 0.03, 95% CI: 0.92–1.02, *P* < 0.001) (Supplementary Figure 8). Detailed subgroup analyses for both CD and UC cohorts are described in Supplementary Figures 7 and 8.

## Discussion

In this study, we demonstrate that serum Eotaxin-1, SAA, IL-6, IL-8, IL-17A, and TNF-α are better predictors of endoscopic disease activity in IBD than the routinely applied serum CRP, fecal calprotectin levels and HBI or SCCAI scores. A combined panel of Eotaxin-1, SAA, IL-6, and IL-8 showed the best prediction of the actual mucosal status in IBD with a sensitivity of 90.7% and specificity of 68.4%. Furthermore, only a few patients were misclassified as having high endoscopic disease activity, yielding a positive predictive value of 86.7%. The combination of these four inflammatory biomarkers demonstrated higher discriminative value regarding endoscopic disease activity in IBD than routinely applied measures of disease activity (i.e., serum CRP, fecal calprotectin levels, and clinical disease indices [HBI/SCCAI]).

All biomarkers that were found to be predictive for endoscopically confirmed disease activity are involved in the pathogenesis of IBD. Eotaxin-1 (CCL11) is a selective chemoattractant and important in the activation and recruitment of eosinophils to the lamina propria of the gut (29). Eotaxin-1 levels have been shown to be elevated in the serum of patients with (active) IBD (30–33). In our study, however, we found higher serum Eotaxin-1 concentrations in UC as compared to CD. Remarkably, serum levels were generally reduced in CD as compared to healthy controls, though there was a large variation in Eotaxin-1 levels in this patient group (Figure 1). Despite this, we observed a clear positive correlation between inflammatory activity in the composite IBD endoscopy score and serum levels of Eotaxin-1 (Supplementary Figure 1). Moreover, serum Eotaxin-1 showed discriminative value for differentiating IBD patients having either remissive or mild disease from patients with moderate or severe endoscopic disease activity (Figure 3). These findings of correlations between serum Eotaxin-1 levels and disease activity corroborate previous observations in human IBD and experimental colitis models that suggested that the eosinophil-selective chemokine Eotaxin-1 associates with disease pathogenesis (34, 35). Eotaxin-1 is produced by intestinal epithelial cells, endothelial cells and macrophages under the influence of several other cytokines that are involved in IBD disease activity, such as IL-17A (36–39).

Serum amyloid A (SAA) was also predictive for IBD disease activity. SAA is an apolipoprotein of high-density lipoproteins (HDL) and belongs to the family of acute-phase reactants. It is produced by the liver upon enhanced serum levels of pro-inflammatory cytokines, such as TNF-α and IL-6, and is enhanced in several chronic inflammatory diseases (40, 41). Previously, it was demonstrated that circulating IL-6 and SAA are useful indicators of disease activity in IBD (42). In contrast to the pro-inflammatory nature of most of the studied cytokines, it is unknown whether SAA contributes to inflammation. The positive correlation with disease activity suggests a pro-inflammatory function, but recently it was also shown that SAA may protect the epithelial barrier by stimulating protective and anti-inflammatory IL-22-producing neutrophils (43). Irrespective of its role in disease development, SAA has been shown to be the most sensitive acute-phase protein in IBD (when compared to other acute phase proteins, such as alpha-1-antichymotrypsin (alpha-1-ACT) and alpha-1-acid glycoprotein (alpha-1-AGP), or even CRP) (44). Therefore, SAA may be of added value as inflammatory biomarker in monitoring the acute-phase reaction, besides CRP (45).

IL-6 was also part of the selected combination of predictive inflammatory biomarkers. IL-6 is one of the most ubiquitously present and pleiotropic cytokines that is involved in most (chronic) inflammatory diseases, including IBD (46). IL-6 can change the balance of effector CD4^+^ T-cell subsets. It is produced by innate immune cells, such as macrophages, neutrophils and mast cells, and forms a bridge between the innate and the adaptive immune system. Upon acute inflammatory events, IL-6 is recognized as important stimulator of acute-phase reactant production in the liver, including CRP. In IBD, the importance of IL-6 is highlighted by the fact that serum concentrations rise concurrently with increasing inflammatory disease activity, as well as elevated soluble receptor complexes (sIL-6R/IL-6) that can bind to and activate IL-6R-lacking immune cells (trans signaling), contributing to chronic mucosal inflammation (47). Pro-inflammatory actions of IL-6 have been demonstrated to predominantly occur via trans signaling, which is strongly associated with the development of and sustainment of intestinal inflammation in IBD (48–50). Here, IL-6 levels fairly accurately differentiated between high and low endoscopic disease activity. As a result, serum IL-6 levels made a substantial contribution to the predictive power of the final biomarker combination.

IL-8 is known as an important neutrophil chemoattractant, modulating recruitment, and degranulation of neutrophils located in the intestinal mucosa (51). Previously, it was demonstrated that serum IL-8 levels are elevated in active IBD, most prominently in UC, as compared to healthy subjects (52). In line, we found significantly elevated serum concentrations of IL-8 in UC compared to CD and healthy controls. Therefore, IL-8 is suggested to be a key factor in the process of neutrophil-mediated intestinal inflammation in active UC. Previously, it was shown that mucosal IL-8 levels can predict future disease relapse in patients with quiescent UC (53). Moreover, serum IL-8 levels present high accuracy in differentiating IBD from irritable bowel syndrome (IBS) patients (54). In this respect, IL-8 might be particularly helpful in identifying an acute disease exacerbation, irrespective of the often non-specific clinical presentation.

Currently, disease activity in IBD is clinically assessed by evaluating a combination of symptoms (quantified with clinical risk scoring methods), biochemical measures such as serum CRP and fecal calprotectin, and ultimately endoscopic evaluation. However, the clinical scoring methods, such as the Harvey Bradshaw Index (HBI) or the Simple Clinical Colitis Activity Index (SCCAI) correlate poorly with endoscopic disease activity (24, 25, 55–57). Our results are in line with these studies, since only serum IL-6 levels correlated significantly with the clinical disease indices in our cohort. Moreover, associations between serum CRP and fecal calprotectin and endoscopic disease activity in IBD appear inconsistent (58). Despite this, these parameters are the most frequently-used non-invasive biomarkers analyzed to monitor disease activity in IBD (14, 23, 59). However, several studies have shown that one single biomarker is unlikely to accurately predict the mucosal status in IBD, given its complex immunological pathogenesis (10, 11, 14, 17, 60). Endoscopic remission is the ultimate goal and measure of therapeutic efficacy in IBD. Additional non-invasive markers are needed to be able to accurately represent endoscopic remission (61, 62). Previous studies have developed disease activity indices reflecting mucosal status, based on clinical characteristics and standard laboratory measurements, but few included inflammatory biomarkers as we investigated in this study (63). Incorporation of such inflammatory biomarkers in existing prediction models or disease indices may contribute to establishing an immunology-based prediction model for endoscopic mucosal status in IBD.

An important strength of the present study is the comprehensive analysis of a selected panel of serum inflammatory biomarkers using an electrochemiluminescence (ECL) assay. Using this highly-sensitive, validated detection method of serum inflammatory biomarkers, we were able to establish serum biomarker concentrations with a broad dynamic range of detection. However, biomarker concentrations were not within the detection range in a small number of samples (6.0%) and were excluded from the analyses. In order to determine whether this may skew the interpretation of our results, we performed a full statistical analysis on a dataset where missing values were replaced by the lower limit of detection (LLoD) or upper limit of detection (ULoD) as indicated by the signals obtained in the ECL assay. Importantly, these analyses further confirmed the final prediction model.

In an earlier study, we found correlations between several serum inflammatory cytokines in CD and fecal calprotectin levels, where we observed positive correlations for Th1- and Th17- associated serum cytokines (including CRP, SAA, and IL-6) and fecal calprotectin levels (22). However, that study was limited by a relatively small cohort of CD patients and the absence of endoscopic results, which prevented us from establishing correlations with IBD disease activity. Likewise, the current study has also some limitations. For instance, a larger cohort would have allowed us to predict endoscopic disease activity using the pre-defined categories as outcome parameter with values ranging from 0 to 3. In this respect, the inclusion of more IBD patients with endoscopic remission could have enabled us to assess the predictive accuracy of a combination of inflammatory biomarkers between the quiescent and active state of the disease and establish clinically useful cut-offs. Similarly, a greater sample size would have resulted in more reliable subgroup analyses for CD and UC. Moreover, this would have provided us with the ability to reliably adjust for confounding variables (e.g., medication use, co-morbidity or acute inflammatory events). Lastly, shortening of the time interval between endoscopy and serum sample collection would have improved the reliability of our results.

Our results demonstrate that a combination of serum inflammatory biomarkers has the potential to differentiate between IBD patients with varying degrees of endoscopic disease activity in a minimally invasive manner. The panel of four biomarkers described in this study has a high accuracy, and it is important now to externally validate this combined array of biomarkers in another IBD cohort. As such, the development of a minimally invasive multi-marker serum test may be particularly clinically relevant as the discrimination between varying degrees of endoscopically active disease may help in guiding therapeutic decision making and adjusting medical therapy (61). Furthermore, endoscopic remission or ‘mucosal healing' is increasingly recognized as important therapeutic endpoint in clinical trials (64). Since prediction of endoscopic disease activity may be found in a combined set of serum biomarkers, this study is also aimed to trigger avenues for future research that further evaluate the potential of a serum biomarker panel to represent disease activity in IBD. Additionally, since cytokines play a pivotal role in the immunopathogenesis of IBD, it is interesting to analyze the effect of induction therapy on serum inflammatory status in relation to endoscopic remission in IBD. Future studies are warranted that focus on the diagnostic potential of this distinct inflammatory biomarker profile in predicting response to (biological) therapy in IBD.

In conclusion, the panel of four serum inflammatory biomarkers identified in this study shows a predictive value of endoscopic disease activity in IBD that is much better than current routine laboratory tests. SAA, Eotaxin-1, IL-6, IL-8, IL-17A, and TNF-α all individually showed better predictive performances compared to CRP, fecal calprotectin and HBI/SCCAI scores. The best prediction of luminal disease activity was observed when SAA, IL-6, IL-8, and Eotaxin-1 were combined, which, as a relatively small panel of biomarkers, harbors great potential to improve monitoring of intestinal inflammatory activity and therapeutic efficacy in IBD.

## Data Availability Statement

The datasets generated for this study are available on request to the corresponding author.

## Ethics Statement

This study was approved by the Institutional Review Board (Dutch: Medisch Ethische Toetsingscommissie [METc]) of the University Medical Center Groningen, Groningen, the Netherlands. All patients provided written informed consent to participate in this study.

## Author Contributions

AB, JM, RG, KF, and GD were involved in conceptualization and study design. GD and KF were responsible for funding acquisition and resources. GD obtained approval from the medical ethical board. AR, JM, RG, TB, MB-H, JH, BJ, HD, EF, RS, MV, RW, KF, and GD collected all study data. AB performed data curation, data analysis and visualization. AB, JM, and RG wrote the first draft of the manuscript. PV, KF, and GD contributed to results interpretation and critically reviewed the manuscript. All authors contributed to manuscript revision and read and approved the final version of the manuscript.

### Conflict of Interest

The authors declare that the research was conducted in the absence of any commercial or financial relationships that could be construed as a potential conflict of interest.

## References

[1] Abraham C Cho JH. Inflammatory bowel disease. N Engl J Med. (2009) 361:2066–78. 10.1056/NEJMra0804647 19923578 PMC3491806

[2] Sartor RB. Mechanisms of disease: pathogenesis of Crohn's disease and ulcerative colitis. Nat Clin Pract Gastroenterol Hepatol. (2006) 3:390–407. 10.1038/ncpgasthep0528 16819502

[3] Schroeder KW Tremaine WJ Ilstrup DM. Coated oral 5-aminosalicylic acid therapy for mildly to moderately active ulcerative colitis. A randomized study. N Engl J Med. (1987) 317:1625–9. 10.1056/NEJM198712243172603 3317057

[4] Daperno M D'Haens G Van Assche G Baert F Bulois P Maunoury V. Development and validation of a new, simplified endoscopic activity score for Crohn's disease: the SES-CD. Gastrointest Endosc. (2004) 60:505–12. 10.1016/S0016-5107(04)01878-4 15472670

[5] Lichtenstein GR Yan S Bala M Hanauer S. Remission in patients with Crohn's disease is associated with improvement in employment and quality of life and a decrease in hospitalizations and surgeries. Am J Gastroenterol. (2004) 99:91–6. 10.1046/j.1572-0241.2003.04010.x 14687148

[6] Ramadas AV Gunesh S Thomas GA Williams GT Hawthorne AB. Natural history of Crohn's disease in a population-based cohort from Cardiff (1986–2003): a study of changes in medical treatment and surgical resection rates. Gut. (2010) 59:1200–6. 10.1136/gut.2009.202101 20650924

[7] Rutgeerts P Vermeire S Van Assche G Mucosal healing in inflammatory bowel disease: impossible ideal or therapeutic target? Gut. (2007) 56:453–5. 10.1136/gut.2005.088732 17369375 PMC1856849

[8] Rutgeerts P Feagan BG Lichtenstein GR Mayer LF Schreiber S Colombel JF. Comparison of scheduled and episodic treatment strategies of infliximab in Crohn's disease. Gastroenterology. (2004) 126:402–13. 10.1053/j.gastro.2003.11.014 14762776

[9] Benitez JM Meuwis MA Reenaers C Van Kemseke C Meunier P Louis E. Role of endoscopy, cross-sectional imaging and biomarkers in Crohn's disease monitoring. Gut. (2013) 62:1806–16. 10.1136/gutjnl-2012-303957 24203056

[10] Minderhoud IM Samsom M Oldenburg B. What predicts mucosal inflammation in Crohn's disease patients? Inflamm Bowel Dis. (2007) 13:1567–72. 10.1002/ibd.20233 17663422

[11] Cellier C Sahmoud T Froquel E Adenis A Belaiche J Bretagne JF Correlations between clinical activity, endoscopic severity, and biological parameters in colonic or ileocolonic Crohn's disease. A prospective multicentre study of 121 cases. The Groupe d'Études Thérapeutiques des Affections Inflammatoires Digestives. Gut. (1994) 35:231–5. 10.1136/gut.35.2.231 7508411 PMC1374499

[12] Jones J Loftus EV Jr Panaccione R Chen LS Peterson S McConnell J. Relationships between disease activity and serum and fecal biomarkers in patients with Crohn's disease. Clin Gastoenterol Hepatol. (2008) 6:1218–24. 10.1016/j.cgh.2008.06.010 18799360

[13] Carlsen K Riis LB Elsberg H Maagaard L Thorkilgaard T Sørbye SW. The sensitivity of fecal calprotectin in predicting deep remission in ulcerative colitis. Scand J Gastroenterol. (2018) 53:825–30. 10.1080/00365521.2018.1482956 29968483

[14] Lewis JD. The utility of biomarkers in the diagnosis and therapy of inflammatory bowel disease. Gastroenterology. (2011) 140:1817–26. 10.1053/j.gastro.2010.11.058 21530748 PMC3749298

[15] Langhorst J Elsenbruch S Koelzer J Rueffer A Michalsen A Dobos GJ Noninvasive markers in the assessment of intestinal inflammation in inflammatory bowel disease: performance of fecal lactoferrin, calprotectin, and PMN-elastase, CRP, and clinical indices. Am J Gastroenterol. (2008) 103:162–9. 10.1111/j.1572-0241.2007.01556.x 17916108

[16] Vermeire S Van Assche G Rutgeerts P. Laboratory markers in IBD: useful, magic, or unnecessary toys? Gut. (2006) 55:426–31. 10.1136/gut.2005.069476 16474109 PMC1856093

[17] Park JH Peyrin-Biroulet L Eisenhut M Shin JI. IBD immunopathogenesis: a comprehensive review of inflammatory molecules. Autoimmun Rev. (2017) 16:416–24. 10.1016/j.autrev.2017.02.013 28212924

[18] Neurath MF. Cytokines in inflammatory bowel disease. Nat Rev Immunol. (2014) 14:329–42. 10.1038/nri3661 24751956

[19] Strober W Fuss IJ Blumberg RS. The immunology of mucosal models of inflammation. Annu Rev Immunol. (2002) 20:495–549. 10.1146/annurev.immunol.20.100301.064816 11861611

[20] Brand S. Crohn's disease: Th1, Th17 or both? The change of a paradigm: new immunological and genetic insights implicate Th17 cells in the pathogenesis of Crohn's disease. Gut. (2009) 58:1152–67. 10.1136/gut.2008.163667 19592695

[21] Sands BE. Biomarkers of inflammation in inflammatory bowel disease. Gastroenterology. (2015) 149:1275–85. 10.1053/j.gastro.2015.07.003 26166315

[22] Bourgonje AR von Martels JZH de Vos P Faber KN Dijkstra G. Increased fecal calprotectin levels in Crohn's disease correlate with elevated serum Th1- and Th17-associated cytokines. PLoS ONE. (2018) 13:e0193202. 10.1371/journal.pone.0193202 29466406 PMC5821357

[23] Lennard-Jones JE. Classification of inflammatory bowel disease. Scand J Gastroenterol Suppl. (1989) 170:2–6. 10.3109/00365528909091339 2617184

[24] Harvey RF Bradshaw JM. A simple index of Crohn's-disease activity. Lancet. (1980) 1:514. 10.1016/S0140-6736(80)92767-1 6102236

[25] Walmsley RS Ayres RC Pounder RE Allan RN. A simple clinical colitis activity index. Gut. (1998) 43:29–32. 10.1136/gut.43.1.29 9771402 PMC1727189

[26] Schoepfer AM Beglinger C Straumann A Trummler M Vavricka SR Bruegger LE. Fecal calprotectin correlates more closely with the Simple Endoscopic Score for Crohn's disease (SES-CD) than CRP, blood leukocytes, and the CDAI. Am J Gastroenterol. (2010) 105:162–9. 10.1038/ajg.2009.545 19755969

[27] Rispo A Testa A De Palma GD Donetto S Diaferia M Musto D. Different profile of efficacy of thiopurines in Ulcerative Colitis and Crohn's disease. Inflamm Bowel Dis. (2015) 21:2570–5. 10.1097/MIB.0000000000000538 26222340

[28] DeLong ER DeLong DM Clarke-Pearson DL. Comparing the areas under two or more correlated receiver operating characteristic curves: a nonparametric approach. Biometrics. (1988) 44:837–45. 10.2307/2531595 3203132

[29] Davoine F Lacy P. Eosinophil cytokines, chemokines, and growth factors: emerging roles in immunity. Front Immunol. (2014) 5:570. 10.3389/fimmu.2014.00570 25426119 PMC4225839

[30] Chen W Paulus B Shu D Wilson Chadwick V. Increased serum levels of eotaxin in patients with inflammatory bowel disease. Scand J Gastroenterol. (2001) 36:515–20. 10.1080/003655201750153377 11346206

[31] Mir A Minquez M Tatay J Pascual I Peña A Sanchiz V. Elevated serum eotaxin levels in patients with inflammatory bowel disease. Am J Gastroenterol. (2002) 97:1452–7. 10.1111/j.1572-0241.2002.05687.x 12094864

[32] Coburn LA Horst SN Chaturvedi R Brown CT Allaman MM Scull BP. High-throughput multi-analyte Luminex profiling implicates eotaxin-1 in ulcerative colitis. PLoS ONE. (2013) 8:e82300. 10.1371/journal.pone.0082300 24367513 PMC3867379

[33] Korolkova OY Myers JN Pellom ST Wang L M'Koma AE. Characterization of serum cytokine profile in predominantly colonic inflammatory bowel disease to delineate ulcerative and Crohn's colitides. Clin Med Insights Gastroenterol. (2015) 8:29–44. 10.4137/CGast.S20612 26078592 PMC4459555

[34] Waddell A Ahrens R Steinbrecher K Donovan B Rothenberg ME Munitz A. Colonic eosinophilic inflammation in experimental colitis is mediated by Ly6C(high) CCR2(+) inflammatory monocyte/macrophage-derived CCL11. J Immunol. (2011) 186:5993–6003. 10.4049/jimmunol.1003844 21498668 PMC3423906

[35] Hogan SP Mishra A Brandt EB Royalty MP Pope SM Zimmermann N. A pathological function for eotaxin and eosinophils in eosinophilic gastrointestinal inflammation. Nat Immunol. (2001) 2:353–60. 10.1038/86365 11276207

[36] Saleh A Shan L Halayko AJ Kung S Gounni AS. Critical role for STAT3 in IL-17A-mediated CCL11 expression in human airway smooth muscle cells. J Immunol. (2009) 182:3357–65. 10.4049/jimmunol.0801882 19265112

[37] Rahman MS Yamasaki A Yang J Shan L Halayko AJ Gounni AS. IL-17A induces eotaxin-1/CC chemokine ligand 11 in human airway smooth muscle cells: role of MAPK (Erk1/2, JNK, and p38) pathways. J Immunol. (2006) 177:4064–71. 10.4049/jimmunol.177.6.4064 16951370

[38] Garcia-Zepeda EA Rothenberg ME Ownbey RT Celestin J Leder P Luster AD. Human eotaxin is a specific chemoattractant for eosinophil cells and provides a new mechanism to explain tissue eosinophilia. Nat Med. (1996) 2:449–56. 10.1038/nm0496-449 8597956

[39] Mishra A Hogan SP Lee JJ Foster PS Rothenberg ME. Fundamental signals that regulate eosinophil homing to the gastrointestinal tract. J Clin Invest. (1999) 103:1719–27. 10.1172/JCI6560 10377178 PMC408388

[40] De Beer FC Mallya RK Fagan EA Lanham JG Hughes GR Pepys MB. Serum amyloid-A protein concentration in inflammatory diseases and its relationship to the incidence of reactive systemic amyloidosis. Lancet. (1982) 2:231–4. 10.1016/S0140-6736(82)90321-X 6124669

[41] Benditt EP Hoffman JS Eriksen N Parmelee DC Walsh KA. SAA, an apoprotein of HDL: its structure and function. Ann N Y Acad Sci. (1982) 389:183–9. 10.1111/j.1749-6632.1982.tb22136.x 7046575

[42] Niederau C Backmerhoff F Schumacher B Niederau C. Inflammatory mediators and acute phase proteins in patients with Crohn's disease and ulcerative colitis. Hepatogastroenterology. (1997) 44:90–107. 9058126

[43] Zhang G Liu J Wu L Fan Y Sun L Qian F. Elevated expression of serum amyloid A 3 protects colon epithelium against acute injury through TLR2-dependent induction of neutrophil IL-22 expression in a mouse model of colitis. Front Immunol. (2018) 9:1503. 10.3389/fimmu.2018.01503 30008720 PMC6033967

[44] Chambers RE Stross P Barry RE Whicher JT. Serum amyloid A protein compared with C-reactive protein, alpha-1-antichymotrypsin and alpha 1-acid glycoprotein as a monitor of inflammatory bowel disease. Eur J Clin Invest. (1987) 17:460–7. 10.1111/j.1365-2362.1987.tb01143.x 3121351

[45] Plevy S Silverberg MS Lockton S Stockfisch T Croner L Stachelski J. Combined serological, genetic, and inflammatory markers differentiate non-IBD, Crohn's disease, and ulcerative colitis patients. Inflamm Bowel Dis. (2013) 19:1139–48. 10.1097/MIB.0b013e318280b19e 23518807 PMC3792797

[46] Hunter CA Jones SA. IL-6 as a keystone cytokine in health and disease. Nat Immunol. (2015) 16:448–57. 10.1038/ni.3153 25898198

[47] Atreya R Mudter J Finotto S Müllberg J Jostock T Wirtz S Blockade of interleukin 6 trans signaling suppresses T-cell resistance against apoptosis in chronic intestinal inflammation: evidence in Crohn's disease and experimental colitis *in vivo*. Nat Med. (2000) 6:583–8. 10.1038/75068 10802717

[48] Chalaris A Schmidt-Arras D Yamamoto K Rose-John S. Interleukin-6 trans-signaling and colonic cancer associated with inflammatory bowel disease. Dig Dis. (2012) 30:492–9. 10.1159/000341698 23108305

[49] Rose-John S Mitsuyama K Matsumoto S Thaiss WM Scheller J. Interleukin-6 trans-signaling and colonic cancer associated with inflammatory bowel disease. Curr Pharm Des. (2009) 15:2095–103. 10.2174/138161209788489140 19519447

[50] Allocca M Jovani M Fiorino G Schreiber S Danese S. Anti IL-6 treatment for inflammatory bowel diseases: next cytokine, next target. Curr Drug Targets. (2013) 14:1508–21. 10.2174/13894501113146660224 24102406

[51] Harada A Sekido N Akahoshi T Wada T Mukaida N Matsushima K. Essential involvement of interleukin-8 (IL-8) in acute inflammation. J Leukoc Biol. (1994) 56:559–64. 10.1002/jlb.56.5.559 7964163

[52] Mahida YR Ceska M Effenberger F Kurlak L Lindley I Hawkey CJ. Enhanced synthesis of neutrophil-activating peptide-1/interleukin-8 in active ulcerative colitis. Clin Sci. (1992) 82:273–5. 10.1042/cs0820273 1312411

[53] Yamamoto T Umegae S Kitagawa T Matsumoto K. Systemic and local cytokine production in quiescent ulcerative colitis and its relationship to future relapse: a prospective pilot study. Inflamm Bowel Dis. (2005) 11:589–96. 10.1097/01.MIB.0000161917.97136.e2 15905707

[54] Neubauer K Matusiewicz M Bednarz-Misa I Gorska S Gamian A Krzystek-Korpacka M. Diagnostic potential of systemic eosinophil-associated cytokines and growth factors in IBD. Gastroenterol Res Pract. (2018) 2018:7265812. 10.1155/2018/7265812 30147719 PMC6083643

[55] Gomes P du Boulay C Smith CL Holdstock G. Relationship between disease activity indices and colonoscopic findings in patients with colonic inflammatory bowel disease. Gut. (1986) 27:92–5. 10.1136/gut.27.1.92 3949241 PMC1433178

[56] Zittan E Kabakchiev B Kelly OB Milgrom R Nguyen GC Croitoru K. Development of the Harvey-Bradshaw Index-pro (HBI-PRO) score to assess endoscopic disease activity in Crohn's disease. J Crohns Colitis. (2017) 11:543–8. 10.1093/ecco-jcc/jjw200 28453763

[57] Baars JE Nuij VJ Oldenburg B Kuipers EJ van der Woude CJ. Majority of patients with inflammatory bowel disease in clinical remission have mucosal inflammation. Inflamm Bowel Dis. (2012) 18:1634–40. 10.1002/ibd.21925 22069022

[58] Solem CA Loftus EV Jr Tremaine WJ Harmsen WS Zinsmeister AR Sandborn WJ. Correlation of C-reactive protein with clinical, endoscopic, histologic, and radiographic activity in inflammatory bowel disease. Inflamm Bowel Dis. (2005) 11:707–12. 10.1097/01.MIB.0000173271.18319.53 16043984

[59] Kapsoritakis AN Koukourakis MI Sfiridaki A Potamianos SP Kosmadaki MG Koutroubakis IE. Mean platelet volume: a useful marker of inflammatory bowel disease activity. Am J Gastroenterol. (2001) 96: 776–81. 10.1111/j.1572-0241.2001.03621.x 11280550

[60] Modigliani R. Endoscopic severity index for Crohn's disease. Gastrointest Endosc. (1990) 36:637. 10.1016/S0016-5107(90)71198-4 2279671

[61] Neurath MF Travis SP. Mucosal healing in inflammatory bowel diseases: a systematic review. Gut. (2012) 61:1619–35. 10.1136/gutjnl-2012-302830 22842618

[62] Florholmen J. Mucosal healing in the era of biologic agents in treatment of inflammatory bowel disease. Scand J Gastroenterol. (2015) 50:43–52. 10.3109/00365521.2014.977943 25523555

[63] Minderhoud IM Steyerberg EW van Bodegraven AA van der Woude CJ Hommes DW Dijkstra G. Predicting endoscopic disease activity in Crohn's disease: a new and validated noninvasive disease activity index (the Utrecht Activity Index). Inflamm Bowel Dis. (2015) 21:2453–9. 10.1097/MIB.0000000000000507 26181428

[64] Walsh A Palmer R Travis S. Mucosal healing as a target of therapy for colonic inflammatory bowel disease and methods to score disease activity. Gastrointest Endosc Clin N Am. (2014) 24:367–78. 10.1016/j.giec.2014.03.005 24975528

