# Supplementary material for: A Combined Set of Four Serum Inflammatory Biomarkers Reliably Predicts Endoscopic Disease Activity in Inflammatory Bowel Disease
Source: Front Med (Lausanne). 2019 Nov 5;6:251. doi: 10.3389/fmed.2019.00251 (PMC6849495; doi:10.3389/fmed.2019.00251)
Supplement: Supplementary file 1 [file Data_Sheet_1.pdf]

Supplementary Material

S1. Baseline demographic, clinical and disease characteristics of the study population

Supplementary Table S1. Baseline demographic and clinical characteristics of the study population (*n* = 138) with comparisons drawn between CD patients (*n* = 64), UC patients (*n* = 54) and healthy controls (HC) (*n* = 20). Data are presented as numbers (proportions, *n* (%)) or mean ± SEM.

Variable	CD	UC	HC	*P*-value
	n = 64	*n* = 54	*n* = 20	
**Age (years)**	43.8 ± 1.8	47.0 ± 2.0	56.1 ± 2.2	**0.004**[†]
**Female sex**	39 (60.9)	26 (48.1)	12 (60.0)	0.348
**BMI (kg/m^2)**	24.0 ± 0.6	26.1 ± 0.8	-	**0.034**
**Active smoking**	19 (32.8)	3 (6.7)	-	**0.005**
**Medication**	39 (60.9)	31 (57.4)	-	0.621
Mesalamine	4 (6.3)	18 (33.3)	-	
Thiopurines/MTX	33 (51.6)	9 (16.7)	-	
Combination therapy	2 (3.1)	4 (7.4)	-	
**Disease duration**	10.8 ± 1.1	7.5 ± 0.7	-	**0.011**
**Prior anti-TNF (no.)**	0.9 ± 0.1	0.8 ± 0.1	-	0.364
**Prior IBD surgery (yes)**	32 (50.0)	4 (7.4)	-	< **0.001**
**Age at diagnosis (Montreal)**				
A1 (< 16 years)	7 (10.9)	4 (7.1)	-	
A2 (17 – 40 years)	44 (68.8)	33 (61.1)	-	
A3 (> 40 years)	13 (20.3)	17 (31.5)	-	
**Disease location CD (Montreal)**				
L1 (ileal)	15 (23.4)	-	-	
L2 (colonic)	8 (12.5)	-	-	
L3 (ileocolonic)	41 (64.1)	-	-	
L4 (upper disease)	0 (0.0)	-	-	
**Perianal disease CD (Montreal)**				
P0 (no)	45 (70.3)	-	-	

P1 (yes)	19 (29.7)	-	-
**Disease extent UC (Montreal)**			
E1 (proctitis)	-	2 (3.7)	-
E2 (left-sided colitis)	-	19 (35.2)	-
E3 (pancolitis)	-	33 (61.1)	-
**Disease behavior CD (Montreal)**			
B1 (non-penetrating, non-stricturing)	25 (39.1)	-	-
B2 (stricturing)	22 (34.4)	-	-
B3 (penetrating)	17 (26.6)	-	-
**Disease severity UC (Montreal)**			
S1 (mild)	-	7 (13.0)	-
S2 (moderate)	-	29 (53.7)	-
S3 (severe)	-	18 (33.3)	-

Differences between two groups were tested using Student's *t*-tests or Mann-Whitney *U*-tests (depending on normality) in case of continuous variables and Fisher's exact tests for categorical variables, as appropriate. [†]Overall *P*-value of one-way analysis of variance (ANOVA) test. *P*-values < 0.05 were considered statistically significant.

Supplementary Table S2. Baseline clinical, biochemical and endoscopic disease activity measures for patients with either CD or UC. Data are presented as numbers (proportions, *n* (%)), mean ± SEM or median (IQR), as appropriate.

Variable	CD	UC	*P*-value
Clinical			
HBI or SCCAI	8 (6 – 11)	6 (4 – 8)	
Biochemical			
Hemoglobin (mmol/l)	7.6 ± 0.1	7.6 ± 0.2	0.768
CRP (mg/l)	11.6 ± 1.8	4.6 ± 0.7	**0.001**
ESR (mm/h)	30.7 ± 3.3	20.5 ± 2.7	**0.021**
WBC (x10^9/l)	8.1 ± 0.5	7.9 ± 0.5	0.822
Thrombocytes (x10^9/l)	326 ± 14	283 ± 9	0.114
Fecal calprotectin (μg/g)	995 ± 141	1,824 ± 239	**0.004**
Endoscopic			
Categories	SES-CD (*n* = 36)	Mayo (*n* = 35)	
0 (Remission)	3 (8.3)	0 (0.0)	
1 (Mild disease)	14 (38.9)	7 (20.0)	
2 (Moderate disease)	13 (36.1)	9 (25.7)	
3 (Severe disease)	6 (16.7)	19 (54.3)	
	Composite IBD endoscopy score (*n* = 71)		
0 (Remission)	3 (4.2)		
1 (Mild disease)	21 (29.6)		
2 (Moderate disease)	22 (31.0)		
3 (Severe disease)	25 (35.2)		

Differences between groups were tested using Mann-Whitney U-tests in case of continuous variables and Fisher's exact tests for categorical variables. *P*-values < 0.05 were considered statistically significant.

Supplementary Table S3. Baseline demographic and clinical characteristics of the subset of IBD patients who underwent endoscopic investigation prior to serum analysis (n = 71).

Variable	CD	UC	*P*-value
	n = 36	*n* = 35	
**Age (years)**	41.3 ± 2.3	44.7 ± 2.6	0.326
**Female sex**	26 (72.2)	16 (45.7)	**0.023**
**BMI (kg/m^2)**	22.4 ± 0.7	25.2 ± 0.8	**0.010**
**Active smoking**	12 (35.3)	2 (7.4)	**0.023**
**Medication**	19 (52.8)	22 (62.9)	
Mesalamine	2 (5.6)	16 (45.7)	
Thiopurines/MTX	16 (44.4)	4 (11.4)	
Combination therapy	1 (2.8)	2 (5.7)	
**Disease duration**	9.3 ± 1.1	7.1 ± 0.8	0.128
**Prior anti-TNF (no.)**	1.2 ± 0.2	0.9 ± 0.1	0.113
**Prior IBD surgery (yes)**	19 (52.8)	0 (0.0)	**< 0.001**
**Age at diagnosis (Montreal)**			
A1 (< 16 years)	4 (11.1)	4 (11.4)	
A2 (17 – 40 years)	24 (66.7)	21 (60.0)	
A3 (> 40 years)	8 (22.2)	10 (28.6)	
**Disease location CD (Montreal)**			
L1 (ileal)	9 (25.0)	-	
L2 (colonic)	3 (8.3)	-	
L3 (ileocolonic)	24 (66.7)	-	
L4 (upper disease)	0 (0.0)	-	
**Perianal disease CD (Montreal)**			
P0 (no)	27 (75.0)	-	
P1 (yes)	9 (25.0)	-	
**Disease extent UC (Montreal)**			
E1 (proctitis)	-	1 (2.9)	
E2 (left-sided colitis)	-	8 (22.9)	
E3 (pancolitis)	-	26 (74.3)	

**Disease behavior CD (Montreal)**		
B1 (non-penetrating, non-stricturing)	15 (41.7)	-
B2 (stricturing)	13 (36.1)	-
B3 (penetrating)	8 (22.2)	-
**Disease severity UC (Montreal)**		
S1 (mild)	-	4 (11.4)
S2 (moderate)	-	21 (60.0)
S3 (severe)	-	10 (28.6)

Data are presented as numbers (proportions, n (%)) or mean ± SEM. Differences between two groups were tested using Student's *t*-tests or Mann-Whitney *U*-tests (depending on normality) in case of continuous variables and Fisher's exact tests for categorical variables, as appropriate. *P*-values < 0.05 were considered statistically significant.

S2. Predicting endoscopic disease activity using inflammatory biomarkers

S2.1 IBD cohort

Distributions of serum concentrations of all biomarkers were compared between IBD patients with binary categorized, composite IBD endoscopic disease activity: remission (0) or mild (1) endoscopic disease activity vs. moderate (2) or severe (3) endoscopic disease activity (**Supplementary Table S4**).

S2.2 CD cohort

In the subgroup analysis of the CD cohort, using the binary ordered Simple Endoscopic Score for CD (SES-CD), patients with high endoscopic disease activity (i.e. moderate or severe) showed significantly increased concentrations of SAA, IFN-γ, IL-6 and IL-17A (**Supplementary Table S5; Supplementary Figure S2**).

Regarding their predictive value for SES-CD scored endoscopic disease activity, serum levels of SAA presented the best discriminative capacity as represented by an AuROC of 0.79 (SE: 0.09, 95% CI: 0.61 – 0.96 , P < 0.01). All other significantly elevated inflammatory biomarkers discriminated well (<u>IFN-γ</u>: AuROC 0.74 (SE: 0.09, 95% CI: 0.56 – 0.92), P < 0.05; <u>IL-6</u>: AuROC 0.71 (SE: 0.09, 95% CI: 0.54 – 0.88), P < 0.05; <u>IL-17A</u>: AuROC 0.78 (SE: 0.08, 95% CI: 0.63 – 0.94), P < 0.01), at least as compared to the standard measures of disease activity (CRP, fecal calprotectin levels and the HBI score) (**Supplementary Figure S3**).

S2.3 UC cohort

In UC patients, using the Mayo endoscopic subscore as predicted outcome of binary categorized endoscopic disease activity, serum levels of IL-6, TNF-α and Eotaxin-1 were significantly increased in moderate-to-severe disease activity as compared to remission or mild disease activity (**Supplementary Table S5; Supplementary Figure S4**).

In ROC analysis, IL-6 demonstrated the highest discriminative ability in predicting binary endoscopic disease activity with an AuROC of 0.82 (SE: 0.14, 95% CI: 0.55 – 1.10, P < 0.05). Predictive performances for TNF-α and Eotaxin-1 were 0.76 (SE: 0.10, 95% CI: 0.57 – 0.95), P < 0.05) and 0.77 (SE: 0.12, 95% CI: 0.53 – 1.00, P < 0.05), respectively. Lastly, SAA showed near-to-significance and had an AuROC of 0.74 (SE: 0.09, 95% CI: 0.56 – 0.92, P = 0.052) (**Supplementary Figure S5**).

Supplementary Table S4. Distributions of serum concentrations of all detected molecules among binary categorized endoscopic disease activity (remission or mild disease (0-1) vs. moderate or severe disease (2-3)) using a composite IBD endoscopy score (CD: SES-CD, UC: Mayo score). Data are presented as median (IQR).

**Detected molecules**	**Remission or mild disease (0-1)**	**Moderate or severe disease (2-3)**	***P*-value**
**Composite Endoscopy Score**	***n* = 24**	***n* = 47**	
**CRP (mg/l)**	3.95 (0.64 – 11.5)	5.82 (1.57 – 16.5)	0.189
**SAA (mg/l)**	3.93 (2.28 – 9.52)	13.6 (4.73 – 52.1)	**0.002**
**IFN-γ (pg/ml)**	6.33 (2.89 – 8.99)	8.29 (4.41 – 14.5)	0.118
**TNF-α (pg/ml)**	2.00 (1.24 – 2.62)	2.30 (1.87 – 3.05)	**0.039**
**IL-6 (pg/ml)**	0.67 (0.30 – 1.41)	0.96 (0.65 – 2.16)	**0.025**
**IL-8 (pg/ml)**	5.39 (3.58 – 8.06)	8.47 (5.46 – 11.7)	**0.005**
**IL-10 (pg/ml)**	0.40 (0.25 – 0.54)	0.48 (0.32 – 1.36)	0.103
**IL-17A (pg/ml)**	1.46 (1.05 – 2.75)	2.90 (1.69 – 4.34)	**0.005**
**Eotaxin-1 (ng/ml)**	0.18 (0.14 – 0.24)	0.27 (0.20 – 0.36)	**0.001**
**Eotaxin-3 (pg/ml)**	19.2 (14.1 – 26.9)	18.4 (11.4 – 22.8)	0.313

Differences between groups were tested using Mann-Whitney U-tests. *P*-values < 0.05 were considered statistically significant (Bonferroni-adjusted).

Supplementary Table S5. Distributions of serum concentrations of all detected molecules among binary categorized endoscopic disease activity (remission or mild disease vs. moderate or severe disease) using the SES-CD for CD and Mayo endoscopic subscore for UC. Data are presented as median (IQR).

Detected molecules	Remission or mild disease (0-1)	Moderate or severe disease (2-3)	*P*-value
**SES-CD**	***n* = 17**	***n* = 19**	
**CRP (mg/l)**	4.04 (1.11 – 13.1)	14.8 (4.86 – 39.3)	0.064
**SAA (mg/l)**	4.17 (2.28 – 11.2)	17.8 (9.76 – 32.7)	**0.009**
**IFN-γ (pg/ml)**	7.44 (4.09 – 10.9)	12.0 (8.68 – 23.1)	**0.020**
**TNF-α (pg/ml)**	2.11 (1.42 – 2.61)	2.15 (1.68 – 2.93)	0.623
**IL-6 (pg/ml)**	0.69 (0.44 – 1.46)	1.58 (0.81 – 2.79)	**0.034**
**IL-8 (pg/ml)**	5.38 (3.72 – 6.60)	6.23 (4.72 – 10.4)	0.084
**IL-10 (pg/ml)**	0.39 (0.24 – 0.44)	0.41 (0.25 – 1.20)	0.525
**IL-17A (pg/ml)**	1.37 (0.92 – 2.65)	3.06 (1.67 – 3.95)	**0.005**
**Eotaxin-1 (ng/ml)**	0.18 (0.15 – 0.24)	0.23 (0.18 – 0.29)	0.159
**Eotaxin-3 (pg/ml)**	16.7 (14.0 – 26.0)	13.8 (9.45 – 23.5)	0.154
**Mayo Score**	***n* = 7**	***n* = 28**	
**CRP (mg/l)**	2.81 (0.54 – 9.65)	3.16 (0.92 – 7.64)	0.680
**SAA (mg/l)**	2.85 (2.07 – 8.88)	13.1 (3.81 – 58.5)	**0.053**
**IFN-γ (pg/ml)**	2.27 (1.62 – 6.24)	5.09 (3.79 – 9.81)	0.088
**TNF-α (pg/ml)**	1.44 (1.07 – 2.68)	2.61 (1.88 – 3.60)	**0.035**
**IL-6 (pg/ml)**	0.23 (0.18 – 1.09)	0.91 (0.54 – 1.70)	**0.024**
**IL-8 (pg/ml)**	6.90 (3.33 – 8.66)	9.05 (5.51 – 13.9)	0.070
**IL-10 (pg/ml)**	0.48 (0.26 – 0.76)	0.52 (0.32 – 1.38)	0.440
**IL-17A (pg/ml)**	2.15 (1.16 – 12.6)	2.71 (1.73 – 4.78)	0.678
**Eotaxin-1 (ng/ml)**	0.16 (0.08 – 0.33)	0.30 (0.25 – 0.40)	**0.035**
**Eotaxin-3 (pg/ml)**	21.3 (14.7 – 36.4)	19.0 (12.6 – 22.6)	0.455

Differences between groups were tested using Mann-Whitney U-tests. *P*-values < 0.05 were considered statistically significant.

S3. Best predictive combinations of inflammatory biomarkers

S3.1 Alternative best predictive combination by replacing SAA with CRP

Interestingly, regarding the final best combination of inflammatory biomarkers as represented by the combination of SAA, IL-6, IL-8 and Eotaxin-1 (**Figure 4A**), SAA could be replaced by CRP levels without losing overall classification performance (**Supplementary Figure S6**) (AuROC 0.84, SE: 0.05, 95% CI: 0.73 – 0.94, $P < 0.0001$, $n = 69$).

S3.2 Best predictive combinations for CD and UC cohorts

In the CD cohort, no combination of inflammatory biomarkers showed better predictive performance than serum levels of SAA with an AuROC of 0.79 (SE: 0.09, 95% CI: 0.61 – 0.96, $P < 0.01$), which showed higher discriminative capacity as compared to the standard measures of disease activity (CRP, fecal calprotectin levels and HBI score) (**Supplementary Figure S7**). Serum SAA levels showed a maximum sensitivity of 86.7% and specificity of 71.4% in correctly classifying CD patients into either low or high endoscopic disease activity (Youden's index = 0.57). In the UC cohort, the combination of IL-6 and Eotaxin-1 demonstrated significantly improved predictive performance (AuROC 0.97, SE: 0.03, 95% CI: 0.92 – 1.02, $P < 0.001$), definitely as compared to standard measures of disease activity (CRP, fecal calprotectin levels and SCCAI score) (**Supplementary Figure S8**). The combination of IL-6 and Eotaxin-1 presented a maximum sensitivity of 92.9% and specificity of 100% in correctly classifying UC patients into either low or high endoscopic disease activity (Youden's index = 0.93).

Supplementary Figure Legends

Supplementary Figure S1 (A-C). Serum levels of cytokines **(A)** Eotaxin-1, **(B)** IL-8 and **(C)** the acute-phase protein serum amyloid A (SAA) significantly correlate with the endoscopic disease activity as represented by the composite IBD endoscopy score (categories ranging from 0-3 are shown on x-axis). Correlations were established using Spearman's rank correlation coefficient (ρ). $^{*}P < 0.05$. $^{**}P < 0.01$.

Supplementary Figure S2 (A-D). Distributions of serum concentrations of **(A)** interferon-gamma (IFN-γ), **(B)** serum amyloid A (SAA), **(C)** interleukin-6 (IL-6) and **(D)** interleukin-17A (IL-17A), that were significantly different among binary categorized endoscopic disease activity (remission (0) or mild (1) disease vs. moderate (2) or severe (3) disease), using the Simple Endoscopic Score for Crohn's disease (SES-CD). $^{*}P < 0.05$. $^{**}P < 0.01$.

Supplementary Figure S3 (A-D). Discriminative capacity of **(A)** serum amyloid A (SAA), **(B)** interferon-gamma (IFN-γ), **(C)** interleukin-6 (IL-6) and **(D)** interleukin-17A (IL-17A) regarding binary categorized endoscopic disease activity in CD (remission (0) or mild (1) vs. moderate (2) or severe (3) disease) as represented by the area under the receiver operating characteristics curve (AuROC). Of all individual molecules shown, SAA displayed the best discriminative capacity regarding binary ordered SES-CD endoscopic disease activity.

Supplementary Figure S4 (A-D). Distributions of serum concentrations of **(A)** interleukin-6 (IL-6), **(B)** tumor necrosis factor alpha (TNF-α), **(C)** serum amyloid A (SAA) and **(D)** Eotaxin-1, that were (almost) significantly different among binary categorized endoscopic disease activity (remission (0) or mild (1) disease vs. moderate (2) or severe (3) disease) using the Mayo endoscopic subscore for ulcerative colitis. $^{*}P < 0.05$.

Supplementary Figure S5 (A-D). Discriminative capacity of **(A)** interleukin-6 (IL-6), **(B)** tumor necrosis factor alpha (TNF-α), **(C)** serum amyloid A (SAA) and **(D)** Eotaxin-1 regarding binary categorized endoscopic disease activity (remission (0) or mild (1) vs. moderate (2) or severe (3) disease) as represented by areas under the receiver operating characteristics curve (AuROC). Of all individual molecules shown, IL-6 displayed the best discriminative capacity regarding binary ordered Mayo endoscopic disease activity.

Supplementary Figure S6. The combination of C-reactive protein (CRP), interleukin-6 (IL-6), interleukin-8 (IL-8) and Eotaxin-1 showed similar discriminative capacity as compared to the model presented in the main article (**Figure 4A**), but was not primarily presented since solely serum CRP showed bad predictive performance in our study cohort.

Supplementary Figure S7 (A-D). Areas under the receiver operating characteristics curve (AuROC) for **(A)** the best predictive performance as represented by serum amyloid A (SAA) levels (mg/l), **(B)** serum C-reactive protein (CRP) levels (mg/l), **(C)** fecal calprotectin (FC) levels (µg/g) and **(D)** Harvey Bradshaw Index (HBI). The best discriminative performance to predict binary ordered endoscopic disease activity using the SES-CD, is demonstrated by solely serum SAA levels (mg/l).

Supplementary Figure S8 (A-D). Areas under the receiver operating characteristics curve (AuROC) for **(A)** the best predictive performance as represented by the combination of interleukin-6 (IL-6) and Eotaxin-1 levels (pg/ml), **(B)** serum CRP levels (mg/l), **(C)** fecal calprotectin (FC) levels (µg/g) and **(D)** Simple Clinical Colitis Activity Index (SCCAI). The

best discriminative performance to predict binary ordered endoscopic disease activity using the Mayo endoscopic subscore, is demonstrated by the combined serum IL-6 and Eotaxin-1 levels (pg/ml).
